# Connexinopathies: a structural and functional glimpse

**DOI:** 10.1186/s12860-016-0092-x

**Published:** 2016-05-24

**Authors:** Isaac E. García, Pavel Prado, Amaury Pupo, Oscar Jara, Diana Rojas-Gómez, Paula Mujica, Carolina Flores-Muñoz, Jorge González-Casanova, Carolina Soto-Riveros, Bernardo I. Pinto, Mauricio A. Retamal, Carlos González, Agustín D. Martínez

**Affiliations:** Centro Interdisciplinario de Neurociencia de Valparaíso, Instituto de Neurociencia, Facultad de Ciencias, Universidad de Valparaíso, Valparaíso, Chile; Centro de Fisiología Celular e Integrativa, Facultad de Medicina, Clínica Alemana Universidad del Desarrollo, Santiago, Chile

**Keywords:** Connexins, hemichannels, gap junction channels, structure and function, human genetic disease

## Abstract

Mutations in human connexin (Cx) genes have been related to diseases, which we termed connexinopathies. Such hereditary disorders include nonsyndromic or syndromic deafness (Cx26, Cx30), Charcot Marie Tooth disease (Cx32), occulodentodigital dysplasia and cardiopathies (Cx43), and cataracts (Cx46, Cx50). Despite the clinical phenotypes of connexinopathies have been well documented, their pathogenic molecular determinants remain elusive. The purpose of this work is to identify common/uncommon patterns in channels function among Cx mutations linked to human diseases. To this end, we compiled and discussed the effect of mutations associated to Cx26, Cx32, Cx43, and Cx50 over gap junction channels and hemichannels, highlighting the function of the structural channel domains in which mutations are located and their possible role affecting oligomerization, gating and perm/selectivity processes.

## Background

Connexin gap junction channels (GJCs) and hemichannels (HCs) are critical for cellular communication. GJCs allow the intercellular exchange of ions and small molecules (e.g., IP3, cAMP, cGMP, ATP) and diverse metabolites (e.g., sugars, amino acids, glutathione) (reviewed in [1]). The same molecules and ions can pass through HCs, but in this case to take part as autocrine and paracrine signals (reviewed by [2, 3]). Mutations in connexins (Cxs) genes are associated to genetic disorders such as skin abnormalities, cardiopathies, neurodegenerative and developmental diseases, cataracts, and most cases of hereditary deafness (reviewed by [4–6]).

**Fig. 1:**
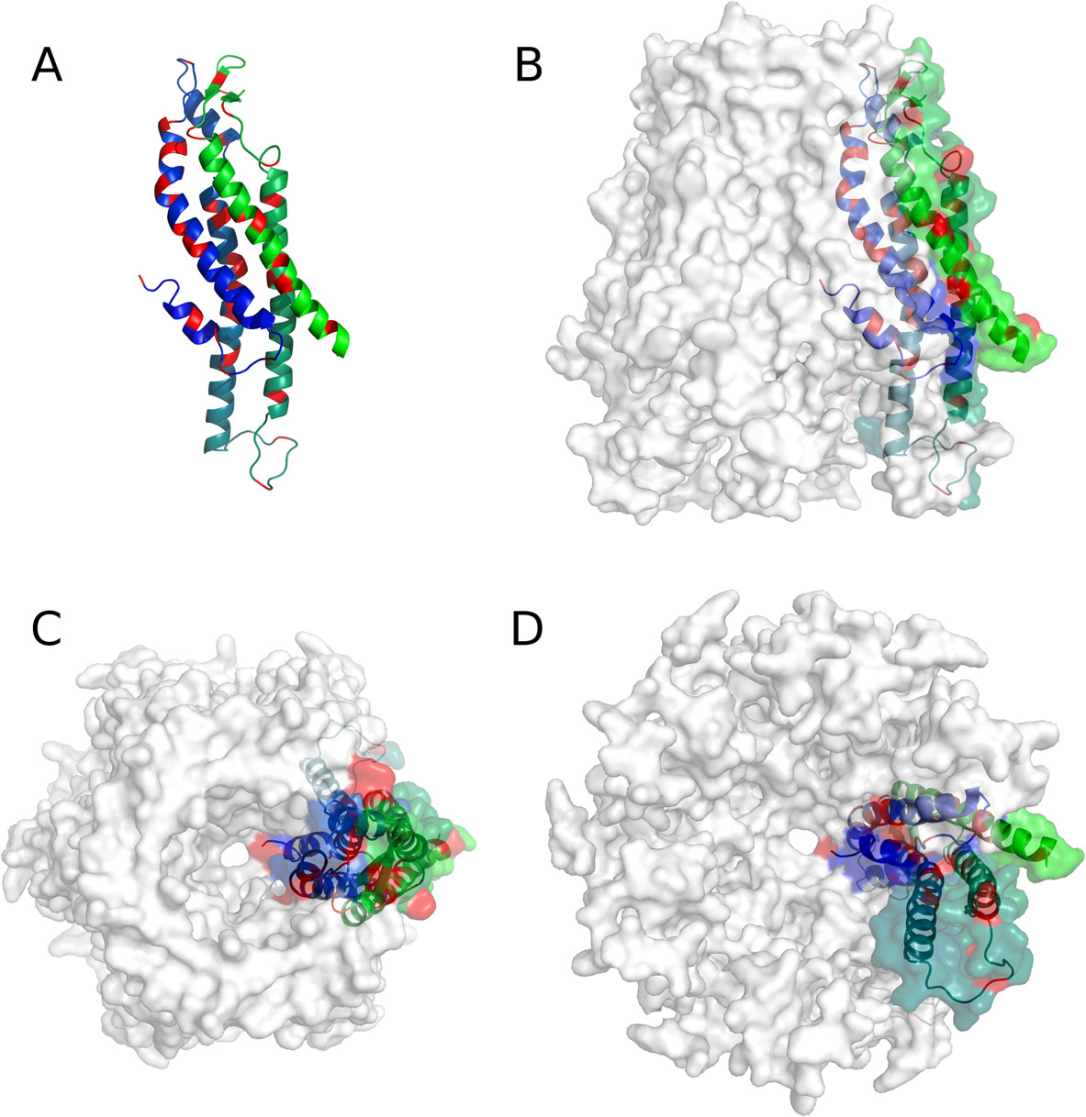
Localization of loss-of-function mutations for Cx26 GJC. **a** Cartoon representation of a Cx26 monomer, colored with a blue-green gradient from the N- to the CT region. Localization of loss-of-function mutations are colored in red. **b** Lateral (**c**) Top (**d**) Bottom view of the same subunit of (**a**), in the context of the HC assemble. The HC surface is transparent and white. The figure was generated with PyMol and edited with Gimp

Each HC is formed by the oligomerization of six Cxs subunits and the end-to-end docking of two HCs forms GJCs. The membrane topology of Cxs includes four transmembrane domains (designated as TM1-TM4) connected by two extracellular loops (ECL) and one intracellular loop (ICL). The amino terminus (NT) and the carboxyl terminus (CT) segments are cytoplasmic (Fig. 1a). Despite Cxs share high homology, there are important differences in the amino acid sequence of the ICL and CT. These segments contain motifs for regulatory kinases and cytoskeletal binding proteins [7, 8]. Oligomerization between suited isoforms also contributes to the assortment of Cx-based channels; for instances, heteromeric GJCs (HCs constituted by more than one Cxs type) and/or heterotypic channels (two homomeric HCs each made by a different Cxs type). These combinations may produce GJCs with particular functional and regulatory properties. Several works pointed out to TM3 in Cx32 [9–11] and Cx43 [12], and TM1 and NT segments in Cx26 [12, 13] as critical to regulate oligomerization of Cxs. In addition, a salt bridge between residues Glu-146 (TM3) and Arg-32 (TM1) in Cx32; and between Lys-22 (TM1) and Glu-209 (TM4) in Cx26, might sustain intraprotomer stability [14]. Nevertheless, the crystal structure of Cx26 showed that the main interactions between protomers occur at the extracellular side of the TM2 and TM4. Moreover, an aromatic cluster formed by the extracellular loops and TM3 also participates in inter-protomer interaction [15]. However, the oligomerization compatibility between Cxs has been associated to specific residues in the NT region [13, 16].

Cxs oligomerize to form a pore whose narrowest part is observed at the ECLs, near the docking zone [15, 17]. As mentioned above, the differences in the amino acid sequences among Cxs may influence the channel properties. It has been proposed that the membrane-spanning regions of Cxs are not only important for intra- and inter- protomer interactions, but they also might determine functional properties such as gating, permeability and the pore’s structure. Concerning the pore composition, there is some controversy about which TMs domains are involved. Some works pointed out the TM3 in Cx32 channels [11, 18] and TM1 in Cx46 channels [19, 20] as principal pore helix components. In support of the role of TM1 as a pore lining segment, it has been proposed that the voltage dependent loop-gating mechanism in the Cx32*Cx43 EL1 chimera (in which the ECL1 of Cx43 replaced the ECL1 of Cx32), involves a rotation of TM1 together with an inward tilt of the six protomers [21]. The 3.5 Å resolution of the Cx26 crystal structure revealed that TM1 is the main constituent of the pore [15] (Fig. 1). The structure also showed that the TM2 lines the pore but in a minor extent, whereas TM3 and TM4 face the hydrophobic membrane environment. The TM1 is tilted, which narrow the pore diameter to 14 Å from the cytoplasmic to the extracellular side of the membrane [15]. More recently, performing molecular dynamic refinements of the crystal structure of Cx26, Kwon and co-workers (2011) [22], shown that the narrow part of the pore could be even smaller.

As it was proposed previously for Cx32 [23] and then confirmed by Maeda and co-workers for Cx26 [15], the Cx-NT domain is located inside the pore, facing the TM1s and forming a funnel like structure that might restricts the pore diameter during gating process [15]. The intra-pore stabilization of the NT is achieved by hydrophobic interactions between residues Trp-3 (NT) and Met-34 (TM1) from neighboring protomers [15]. This interaction was previously proposed by Oshima and co-workers (2007), which found a prominent pore electron-density in the middle of the pore generated by the deafness mutant Cx26M34A. A reduction of this pore electron-density was observed when residues 2-7 (Cx26M34A-del2–7) were deleted [24], confirming the NT as major contributor to the pore occlusion.

Experiments using a chimeric HC of Cx32*Cx43ECL1, have provided more insight about the gating-mechanism of Cx-based channels [21]. In this chimera, the Cys substitution of the Ala residues in positions 40 and 43, located at the TM1/E1 border, form disulphide bonds with adjacent protomers when the cells are bathed in solutions expected to keep HCs closed (5 mM Ca^2+^or 10 µM Cd^2+^). These results strongly suggest a role for these residues in the “loop-gating” mechanism and extracellular Ca^2+^ regulation of HCs [21, 25].

### GJCs and HCs gating regulation

How gating and permeability are regulated in Cxs- based channels is a matter of intense debate. To date, three types of gating mechanism have been proposed: 1) *The NT as a voltage-sensor domain:* that plugs the channel vestibule and contribute to the fast or V_(j)_-gating [15, 26], 2) *The Loop gating*: in which extracellular divalent cations (p.g., Ca^2+^) binds to the extracellular loops and blocks HCs by stabilizing the closed loop gate conformation [25, 27], and 3) *The ball-and-chain model:* which proposes that the CT as part of a ball-and-chain mechanism to regulate the gating of HCs. The last mechanism involves CT conformational rearrangements elicited by voltage or chemical (pH, redox, phosphorylation) stimuli, which promote a link between this segment and the ICL, and regulates the fast V_(j)_-gating mechanism [28–33]. This interaction requires the formation of alpha helical structures on the ICL peptide, in which the CT binds upon intracellular acidification [30].

Considering the relevance of the aforementioned mechanisms for channel function, it is critical to understand how Cxs mutations linked to diseases impair these processes. In the next sections, we describe genetic diseases associated to four Cxs that we used as models for the purpose of this review. For space reason, we did not include information about other important Cxs with mutations associated to disease, like Cx46 mutations linked to congenital cataracts [6, 34] or increased risk to developing diseases, like in polymorphisms in Cx37 genes associated to cardiovascular diseases [35].

### Disease associated to Cx26 mutations

Genetic sensorineural hearing loss is associated mainly to mutations in Cx26 [36] (Table 1). Two clinical phenotypes derive from Cx26 mutations: 1) non-syndromic deafness, in which patients evince moderated to severe deafness with absence of other pathological manifestation; and 2) syndromic deafness, in which profound sensorineural hearing loss is accompanied by a range of severe tissue defects such as the observed in palmoplantar keratoderma [37, 38], keratitis ichthyosis deafness syndrome (KID) [39–42], Vohwinkel syndrome [43], histrix-like ichthyosis with deafness syndrome and Bart-Pumphrey syndrome [44, 45].

**Table 1:** Effect of mutations in Cx26 (GJB2) on the functional state of HCs and GJCs evaluated in a heterologous expression system, the domain that is affected and its phenotype

Domain	Mutation	GJCs Function	HCs Function	Deafness Phenotype
NT	M1V, T8M, G12V [13, 123, 132–136]	(−)	n.d.	NS, Profound, Moderate
	G11E [130, 136, 137]	n.d.	(+)	S, Profound. KID
	G12R^(+*)^, N14K [13, 123, 136, 138]	(−)	(+)	S, Mild, Severe. KID/EKV
	N14D [139]	n.d.	(−)	NS, Moderate
	N14Y^(+*)^ [13, 39, 136]	(−)	(+)	S, Profound. KID
	S17F^(+*)^ [13, 40, 123, 136]	(−)	(−)	S, SNHL. KID
TM1	V27I [140]	Normal	Normal	NS, HL and Normal
	I33T [141]	(−)	n.d.	NS, Severe to Profound
	M34T [36, 115, 142–146]	(−)	(−)	NS, Mild to Moderate; S, Profound. PPK
	V37I, A40G [113, 143, 147–149]	(−)	(−)	NS, Mild-Moderate, Severe
	A40V [124, 136, 150, 151]	Normal	(+)	S, Profound. KID
ECL1	DelE42, D66H [152–159]	(−)	n.d.	S, Profound, Moderate to Profound. PPK
	W44C, W44S, D46E, T55N [142, 143, 152, 153, 159–163]	(−)	n.d.	NS, Severe to Profound, HL, Moderate, Severe
	G45E [124, 130, 150, 164–166]	Normal	(+)	S, Profound. KID
	E47K [164, 167]	(−)	(−)	NS, Severe to Profound
	D50A [168, 169]	n.d.	(+)	S, Profound. KID
	D50N [123, 137, 151, 170–172]	(−)	(+)	S, Profound. KID
	G59V [144, 173]	n.d.	(−)	NS, Profound
	R75Q, R75W [37, 134, 136, 141, 152–154, 174]	(−)	(−)	S, Severe to Profound. PPK
TM2	W77R, F83L, L90V, V95M [37, 135, 142, 143, 147, 173, 175, 176]	(−)	n.d.	NS, Moderate to Profound, Moderate, Profound
	I82M [144, 177]	n.d.	(−)	NS, Profound
	V84L [51, 147, 148, 178, 179]	Normal/No IP3 transfer	n.d	NS. Profound
	T86R, A88S, L90P [132, 143, 144, 147, 160, 180]	(−)	(−)	NS, Profound, Moderate to Profound, Mild to Moderate
	A88V [136, 168, 181]	n.d.	(+)	S, Severe to Profound. KID
ICL	E114G, R127H [115, 140, 144, 173, 178, 182, 183]	(−)	(−)	NS, Severe to Profound, Profound
	DelE120 [141, 143, 147]	(−)	n.d.	NS, Severe to Profound
TM3	R143Q, R153I [133, 152, 153, 183, 184]	(−)	n.d.	NS, Profound
	R143W [133, 144, 178, 185, 186]	(−)	(−)	NS, Profound
ECL2	F161S, P173R, D179N, R165W, W172R, R184P, R184Q [132, 141, 143, 147, 152, 153, 187–190]	(−)	n.d.	NS, HL, Severe to Profound, Profound
	M163L [191]	n.d	(+)	NS, Moderate to Profound
	S183F [136, 192]	(−)	n.d.	S, High Frequency HL. PPK
TM4	M195T, A197S,206S, L214P [133, 135, 190, 193, 194]	(−)	n.d.	NS, HL, Moderate, Profound
	C202F [153, 193, 195]	n.d.	(−)	NS, Mild to Moderate
	I203T, L205V [179, 193, 196]	(−)	(−)	NS, HL, Profound

NS Non-syndromic, S Syndromic, KID Keratitis-Ichthyosis-Deafness, EKV Erythrokeratodermia variabilis, PPK Palmoplantar Keratoderma-deafness, HL Hearing loss. (+*) = Generate gain of HC function when they are coexpressed with wild type Cx26 or Cx43 [13]

(−) = Loss of function. (+) = Gain of function. n.d. = not determined

Among the attempts to identify the pathogenic mechanism of KID syndrome, two transgenic animal models have been developed. They express the Cx26S17F and Cx26G45E mutations in the skin and/or cochlea [46, 47] and exhibit similar phenotypes than humans. Experimental results strongly support that the possible mechanisms in the skin might include the impairment of the epidermal calcium homeostasis and the disruption of the water barrier due to abnormal lipid composition of the stratum corneum [48]. For hearing loss, several hypotheses have been proposed. They include loss of Ca^2+^ homeostasis and ATP release [49, 50], impaired permeability to Ins(1,3,4) P3 [51], loss of the endocochlear potential by deficient K^+^ recycling between the epithelial GJ network and the stria vascularis [52], and developmental malformation or cochlear degeneration induced by massive cell death [53, 54]. For comprehensive reviews see [4, 55].

### Disease associated to Cx32 mutations

Cx32 is expressed in several cell types, including the myelin-forming cells in both the peripheral and central nervous systems (CNS); the Schwann cells and oligodendrocytes, respectively. Mutations in this protein are associated to the most common X-linked inheritance form of the Charcot-Marie-Tooth disease (CMT), a pathology referred as CMT1X that accounts for the 10 % of all the CMT cases [56–58]. Due to its X-linkage, males display moderate to severe symptoms [59, 60], while milder phenotypes are observed in heterozygous females [61, 62].

In the peripheral nervous system, mutations in Cx32 induce progressive muscular atrophy and variable sensory loss, symptoms associated to slow axonal conduction and distal axonal loss [63]. However, prolonged central conductions times of sensory inputs also arise as consequence of Cx32 missense mutations [64–66].

Cx32 localizes in the axonal paranodes and Schmidt-Lantermann incisures [67–69] of the peripheral nerves. Hence, GJC made by this protein do not connect adjacent cells but contiguous loops of non-compact myelin. These channels likely act as a preferential diffusion pathway, significantly decreasing the distance between the nucleus and the adaxonal membrane of the myelin sheaths [67, 70].

The peripheral pathological mechanisms associated to Cx32 mutations possibly involve the loss of function of the GJC (Table 2), either by intracellular retention or the production of channels with aberrant properties [70–72]. This lack of functionality might reduce the transfer of signaling molecules, like cAMP, between the adaxonal portions and the nucleus of the Schwann cell [73].

**Table 2:** Effect of mutations in Cx32 (GJB1) on the functional state of HCs and GJCs evaluated in a heterologous expression system, the domain that is affected and its phenotype

Domain	Mutation	GJCs Function	HCs Function	CMTX Phenotype
NT	W3A, W3S, W3Y, G12S, W13L, V13L, R15W, R22G, R22X [127, 197–203]	(−)	n.d.	Mild to Severe, Severe, Mild to Moderate, Not described
TM1	S26L, M34K, A39V, A40V [70, 71, 204, 205]	(−)	n.d.	Mild, Not Described
	M34T, V35M, V38M [70, 205, 206]	(−)	n.d.	Mild to Moderate, Severe
ECL1	G45E [207]	n.d.	(+)	Not Described
ECL1	C53S, C60F, Y65C, R75P [203, 205, 208–210]	(−)	n.d.	Not Described
	T55I, R75Q, R75W [72, 204, 205, 209, 210],	(−)	n.d.	Mild
TM2	S85C [127, 211]	n.d.	(+)	Severe, Mild
	T86A, T86S, T86N, T87A [70, 212]	(−)	(−)	Not Described, Mild
	H94Y, H94Q [127, 206]	(−)	n.d.	Mild to Moderate
	M93V, V95M [203, 204, 206]	(−)	n.d.	Not Described, Mild to Moderate
ICL	E102G, Null111-116 [71, 198, 202, 213]	(−)	n.d.	Mild, Mild to Moderate
	R107W, R129H [203, 214]	(−)	n.d.	Mild to Moderate, Not described
TM3	V139M, V140E, R142W [127, 197, 209, 215–219]	(−)	n.d.	Mild to Moderate, Mild to Severe, Moderate to Severe
ECL2	L143P, L156R [203, 218]	(−)	n.d.	Mild to Moderate
	R164Q, V181A, E186K [197, 204, 206, 213, 214, 216, 219]	(−)	n.d.	Moderate to Severe
	R164W, P172R, S182T, R183H [72, 198, 203, 204, 206, 208]	(−)	n.d.	Mild to Moderate, Not Described
TM4	G199R, R203C, N205I [203, 205, 206, 214]	(−)	n.d.	Moderate to Severe, Not Described
	E208K, R208K [197, 202, 203, 216, 220, 221]	(−)	(−)	Moderate to Severe
	Y211X [203, 222]	(−)	n.d.	Severe
CT	R215W [206, 209, 221]	(−)	(−)	Mild to Moderate
	C217X [198, 220]	n.d.	(−)	Severe
	R220X [71, 197, 198, 206, 220]	(−)	n.d.	Moderate to Severe
	F235C [126]	n.d.	(+)	Severe
	R265X [198]	(−)	(−)	Severe

(−) = Loss of function. (+) = Gain of function. n.d. = not determined

Furthermore, at least some effects of Cx32 mutations have been associated to a gain of function of the GJC (Table 2). Nevertheless, this is based on indirect electrophysiological studies performed in two patients who do not express Cx32; these patients display visual and auditory evoked responses with normal central conduction times [74, 75]. However, the absence of central functional disruptions in most CMT1X patients and Cx32-KO animals supports the hypothesis of gain of function of GJC in patients where disease also affects CNS [61, 76, 77]. However, further studies about the functional properties of the Cx32 channels are required to support these hypothesis.

### Disease associated to Cx43 mutations

Oculodentodigital Dysplasia (ODDD) is the most important human disease related to Cx43 mutations (Table 3). ODDD is a autosomal inherited developmental disorder affecting face, eyes, teeth and limbs (reviewed in [1, 78]). This pathology was linked to a germ line Cx43 gene (*GJA1*) mutation [79]. The phenotype varies from syndactyli type III alone, to ODDD without syndactyli [80, 81], camptodactyli [79], cardiac impairments, mild cognitive retardation [82] and skeletal abnormalities, which could be associated to impaired osteoblast differentiation [83].

**Table 3:** Effect of mutations in Cx43 (GJA1) on the functional state of HCs and GJCs evaluated in a heterologous expression system, the domain that is affected and its phenotype

Domain	Mutant	GJCs Function	HCs Function	Phenotype
NT	G2V, D3N, W4A, L7V, L11P, S18P [79, 92, 223–225]	(−)	n.d.	*ODDD*
	G12R, Y17S [79, 90, 92, 223, 226–228]	(−)	(−)	*ODDD*
TM1	I31M [91, 229]	(−)	(+)	*ODDD*
	R33X [81, 230]	(−)	n.d.	Small deep-set eyes, syndactyli, dental abnormalities
ECL1	A40V, L90V, F52dup [79, 226, 227, 229, 231]	(−)	(−)	*ODDD*
	E42K [232, 233]	(−)	n.d.	Sudden infant death, lethal ventricular arrhythmias
	Q49K [79, 227, 231, 234]	(−)	n.d.	*ODDD*
	S69P [235]	(−)	n.d.	Nonsyndromic Hearing Loss
	R76H [230, 236]	(−)	n.d.	Hallermann-Streiff syndrome: small stature, hypotrichosis, teeth and skeletal abnormalities
ICL	I130T [79, 89, 226, 227]	(−)	(−)	*ODDD*
	K134E, T154A [89, 226, 236–239]	(−)	n.d.	*ODDD*
	G138R, G143S [79, 89–92]	(−)	(+)	*ODDD*
	H194P [80, 91]	(−)	Normal	*ODDD*
ECL2	R202H, V216L [79, 92, 226, 228, 229, 231]	(−)	n.d.	*ODDD*
TM4	Fs230, Fs260 [92, 240]	(−)	n.d.	*ODDD*
	S272P [232]	Normal	n.d.	Sudden infant death
CT	T326I [235]	(−)	n.d.	Nonsyndromic Hearing Loss
	S364P [98, 241]	(−)	n.d.	Viscero-atrial heterotaxia/heart malformations

(−) = Loss of function. (+) = Gain of function. n.d. = not determined

Currently, over 74 mutations related with ODDD have been reported. However, less than a half of these mutations have been characterized. Missense mutations of Cx43 associated to ODDD are spread through Cx43 amino acid sequence without a clear pattern (Table 3). However, most mutations concentrate in the first half of the protein, with few localized at the CT region (Table 3). The CT domain has several residues that may be phosphorylated, and these allow the regulation of processes like communication, trafficking to the plasma membrane and assembly and degradation of the gap junction protein [84]. The CT also interacts with the ZO-1 [85], v-Scr [86] and other proteins, including cytoskeletal proteins [87].

Several mutations associated to ODDD are located in the ICL region of Cx43 (Table 3), demonstrating the importance of this domain for Cx43 based channels functionality. ICL is critical for both, the pH-mediated gating and the architecture of the channel pore [88]. For example, the ODDD mutant Cx43G138R, which is located in this domain, results in unfunctional GJCs when expressed in N2A cells [89–92]. In contrast, the mutation increases the HC activity determined by ATP release measurements [91]. Moreover, a mouse model carrying this mutation (Cx43G138R) exhibits a phenotype that resembled the observed in humans, i.e., craniofacial alterations, bilateral syndactyli, smaller teeth (microdontia), unspecialized enamel hypoplasia, osteopenia and sparse hair [93].

A principal role of Cx43 GJCs in the myocardium is to allow a rapid and coordinated electrical excitation important for the cardiac-action potential propagation. Cx43 is mainly located at the intercalated discs in the ventricular myocardium. The geometrical arrangement of the discs, as well as the total number of GJCs, seems to be determinant for the characteristic anisotropic conduction of the ventricular myocardium. The atrial myocardium expresses high levels of Cx43 and Cx40 in addition to small quantities of Cx45 [94]. In addition, it has been reported that cells forming the conduction system (responsible for rapid electrical signal localization from the sinoatrial node to the ventricles), express Cx43, Cx45, Cx40, and Cx30 [95, 96]. However, patients with mutation in Cx43 rarely exhibit cardiac problems (Table 3). In addition, congenital heart diseases are not commonly associated to Cx43 mutations [97]. Until now, only a few cases of Cx43 mutations linked to heart diseases have been reported. For example Ser364Pro, which results in viscera atrial heterotaxia [98] restrict GJCs communication in transfected cells. A subsequent work of Thibodeau et al. [99] showed a frameshift mutation in a patient with atrial fibrillation. This modification involves a single nucleotide deletion (c.932delC) with 36 aberrant amino acids with a consecutive stop codon. Interestingly, the mutation was absent in peripheral blood lymphocytes and the immunohistological analysis from left atrial tissue showed areas with normal GJCs localization but at the same time, areas with predominant intracellular retention of Cx.

### Disease associated to Cx50 mutations

Fibers and epithelial cells in the eye lens are connected through Cx50 GJCs [100–102]. This communication is required to maintain the ionic conditions necessary to avoid the formation of cataract [103], a pathology resulting in the opacity of the lens, restricting the amount of light reaching the retina. The Cx50 mutations (Table 4) have been identified in members of families with inherited cataracts. The phenotype may vary across patients, in which missense locations and frame shifts have been commonly identified (reviewed in [6]).

**Table 4:** Effect of mutations in Cx50 (GJA8) on the functional state of HCs and GJCs evaluated in a heterologous expression system, the domain that is affected and its phenotype

Domain	Mutation	GJCs Function	HCs Function	Cataract Phenotype
NT	R23T [242]	(−)	n.d.	Bilateral nuclear
TM1/ECL1	V44A [243]	n.d	(−)	Suture-sparing nuclear
	V44E [110]	(−)	n.d.	Whole lens
	W45S [106, 244]	(−)	(−)	Jellyfish-like appearance, Micro cornea
ECL1	G46V [104, 106]	(+)	(+)	Total
	D47N [110, 117]	(−)	n.d.	Nuclear Pulverulent
	E48K [116, 245]	(−)	Normal	Zonular Nuclear Pulverulent
	S50P [114, 118]	(−)	(−)	Altered fiber cell formation, dense cataract and posterior capsule rupture
TM2	V79L [110]	(−)	n.d.	“Full moon” with Y-suture Opacities
	P88S [34, 246]	(−)	n.d.	Zonular Pulverulent
	P88Q [247]	(−)	n.d.	Lamellar Pulverulent
CT	S276F [248, 249]	(−)	(−)	Nuclear Pulverulent
	Cx50fs [250]	(−)	n.d.	Triangular

(−) = Loss of function. (+) = Gain of function. n.d. = not determined

All Cx50 mutations produce loss of function GJCs, except G46V that produce gain of function GJCs [104]. These mutations could generate both, mislocalization and impaired function of GJCs and HCs (e.g., gating or charge selectivity) [105–107]. At cellular level, it is possible that Cx50 mutations affect the intercellular communication mediated by heteromeric Cx46-Cx50 GJCs. This idea is based on results demonstrating that these Cxs co-localize at GJCs plaques [108–110]. The defective GJCs activity could alter the solute transport between cells and disrupt the Ca^2+^ homeostasis [111, 112]. The abnormal ion transport, especially Na^+^ ions, causes lens swelling and ameliorates the fluid circulation inside the structure. These abnormal processes might affect the nutrient transport and the clearance of noxious metabolites, triggering the cataract formation [112].

### Location of mutations associated to diseases and their functional consequences on GJC and HCs

Taking advantage of the natural occurring mutations in Cxs and previous studies focuses in the effect of disease-associated mutations on the functional state of GJCs and HCs, we looked for similarities and differences between Cxs regarding the positions of mutations associated to the respective diseases and its functional consequences on GJCs and HCs.

Tables summarize experimental results on GJCs and HCs obtained for different Cxs and disease conditions. They show that independent of the disease and Cx, all mutations produce loss of function of the GJCs, which can be partial or total. The decreased GJCs activity can be consequence of reduced amount of channels in the appositional membranes or changes in the functional properties of single channels.

It has been well established that a loss of function of the GJCs elicited by Cx mutations is sufficient to develop pathology. However, it is not clear if the extent of the loss of function is related to the severity of the disease. An institutive reasoning is that there is a good positive correlation between the severity of the Cx-linked disease and the loss of function of the corresponding GJCs. Unfortunately, the experimental data do not support this statement. On one side, positive correlation can be found when the analysis is restricted to some missense non-syndromic Cx26 mutations (V37I and A40G). While these genetic modifications induce GJCs with loss of function (A40G) and active channels with reduced permeability (V37I) [113], they produce a severe deafness phenotype and a milder condition, respectively [4]. However, a clear correlation cannot be established when other mutations are analyzed, such as some Cx32 mutations associated to a mild to moderate (Null111-116) and moderate to severe (R220X) CMTX1 phenotypes. As expected, the permeability of these channels to different dye tracers decreases as the size of the probe increases [114]. However, unlike the channels containing the Null111-116 mutation, permeability of the R220X-Cx32 GJCs to small probes (neurobiotin) is not significantly different from that observed in wild type channels [115]. In the same region (TM1-ECL1) other mutations cause nonfunctional GJCs and HCs (eg. E48K, D47N, S50P) [110, 116–118]. In contrast, Cx50 W45S acts as a dominant negative when co-expressed with Cx50, reducing GJCs coupling between fibers cells [106]. The above evidences suggest that the disease mechanisms might be produced by subtle changes in GJCs permeability, which are impossible to detect by the common electrophysiological and dye coupling methodologies.

**Fig. 2:**
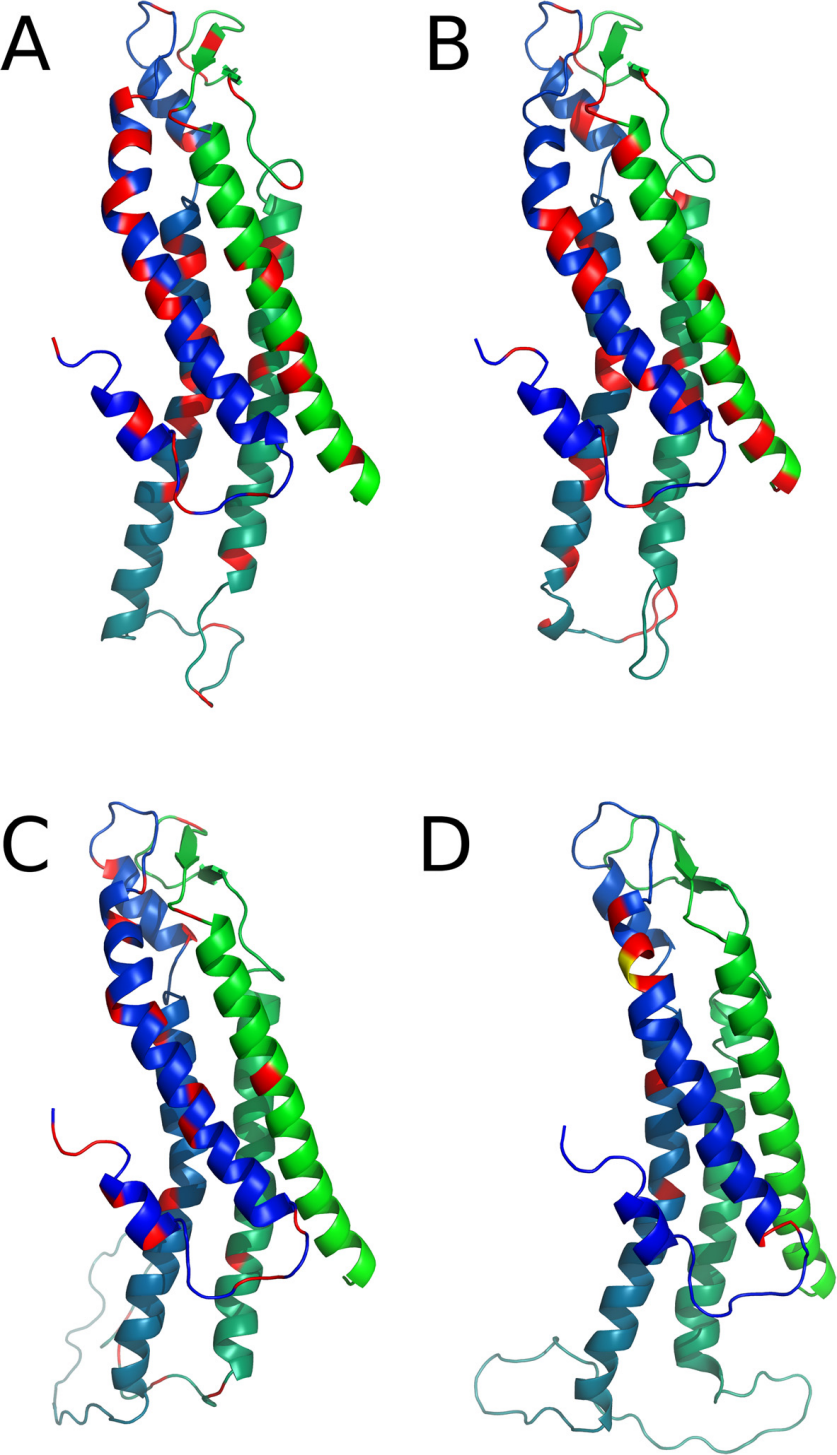
Mutations affecting function of GJCs. Models of single Cxs chains are represented as cartoons, and colored with a blue-green gradient from the N- to the CT region, for (**a**) Cx26 (**b**) Cx32, (**c**) Cx43 and (**d**) Cx50. Positions of loss of function mutations are colored as red and gain of function mutations as yellow. The figure was generated with Pymol and edited with Gimp

**Fig. 3:**
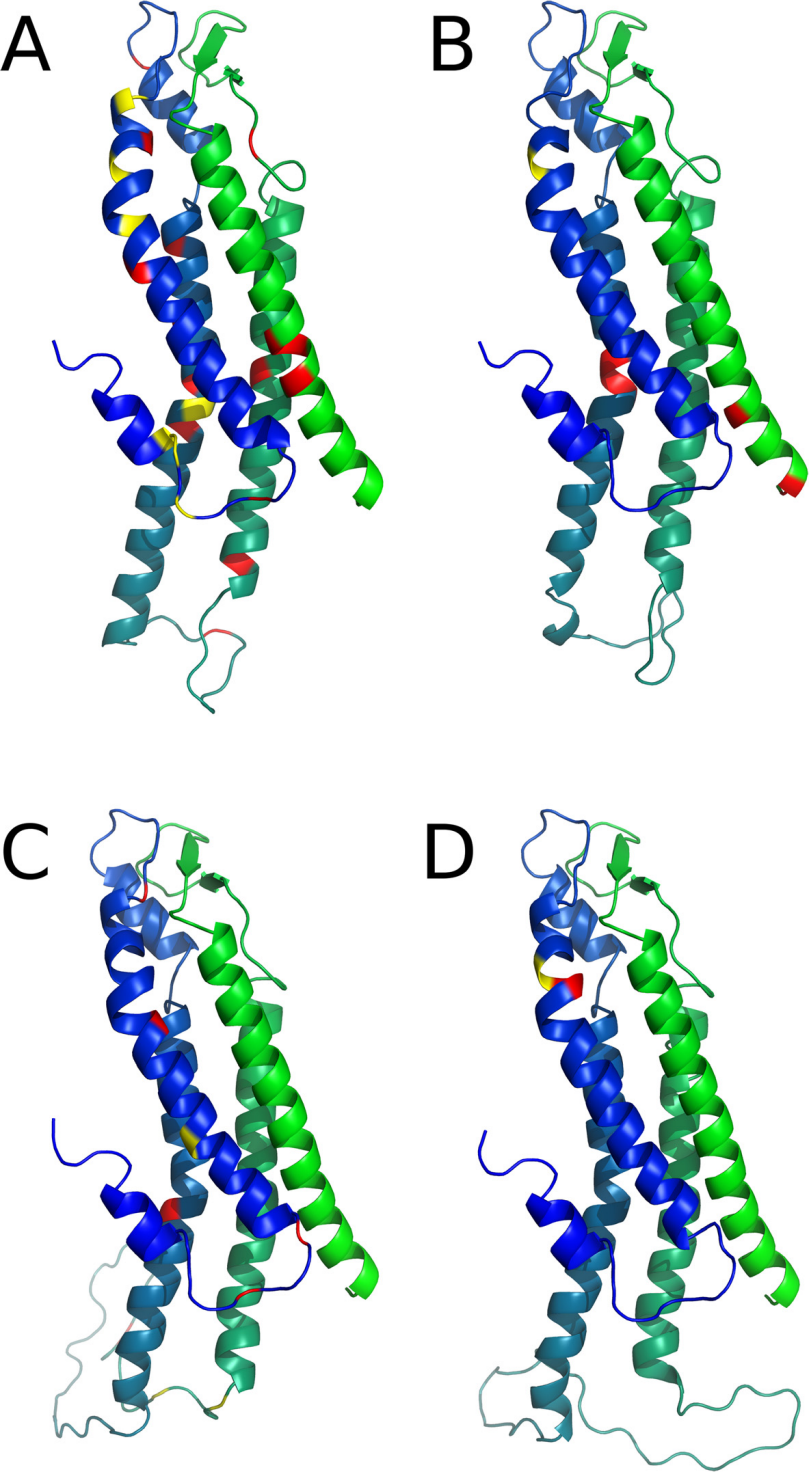
Mutations affecting function of HCs. Models of single Cxs chains are represented as cartoons, and colored with a blue-green gradient from the N- to the CT region, for (**a**) Cx26 (**b**) Cx32, (**c**) Cx43 and (**d**) Cx50. Positions of loss of function mutations are colored as red and gain of function mutations as yellow. The figure was generated with PyMol and edited with Gimp

In order to know the location of mutations in the channel structure, we produced several molecular models of the different Cxs by homology modeling, taking the crystal structure of Cx26 GJC published by Maeda et al., (2009) as template [15]. Due to the lack of experimental structure for human Cx32, Cx43 and Cx50, we generated comparative structural models, using Modeller [119], based on the structure of human Cx26 as a template (pdb: 2ZW3) (Figs. 2 and 3). Missing residues of human Cx26 structure were inserted with Modeller. The backbone of the experimental Cx26 structure was fully conserved. Ten models were generated in each case and those with the lowest discrete optimized protein energy (DOPE) score were selected as the final models. Figure 1 shows the model of a Cx26 monomer in the context of the connexon as well the location of residues mutated in genetic deafness that produce loss of function GJCs. Clearly, although loss of function mutations can be located everywhere in the protomer, they are concentrated from the NT to the TM2 domains (Fig. 1), regions that line the pore and are critical for voltage gating, as we mentioned earlier [120]. Moreover, other mutations in the transmembrane regions seem to be located in protein-protein and protein-lipid interfaces (Fig. 1b, c). Those locations could be important for intra- or inter-protomer interactions [121], which might stabilize the channel or contribute GJCs channel assembly. For Cx32, the pattern for location of mutations that produced loss of function GJCs is very similar to that observed for Cx26 (Fig. 2b), suggesting strong similarities in the structural features between these two Cxs. For Cxs 43 and 50, mutations that produce loss of GJCs function are more restricted. The fact that they localize mostly from NT to ECL1 (Fig. 2c, d) confirms the importance of this region for the channel function in the whole Cx family. However, the ICL Cx43 also presents important amount of mutations producing loss of function GJCs (Table 3).

### Mutations affecting HCs function

The HCs play important role in physiological and pathological conditions since they provide a route for paracrine/autocrine signaling between the cell and the extracellular environment [2, 122]. Hence, a plausible underlying mechanism for connexinopathies is the possibility that some disease condition arise upon HCs dysfunction. For example, aberrant gain of function HCs is associated to syndromic Cx26 mutations that lead to keratitis ichthyosis deafness syndrome (KID) [13, 123, 124]. For the other Cxs (Cx32, Cx43 and Cx50), very few cases have been reported making it difficult to establish a common mechanism of disease (Tables 1, 2, 3 and 4). Exceptions are some mutations in Cx32 (S85C and F235C), which induce aberrant gain of HC activity in CMTX1 [125, 126], which behaves similar to the KID-linked Cx26 mutations, i.e., causing a gain of function of the HCs [125] and a loss of function of the GJCs [127]. Although the S85C mutant induces abnormal HCs opening [128], this mutation has not been associated to any particular severe phenotype of CMTX1 [129].

Most of the mutations eliciting gain of HCs function are clustered exclusively in the pore lining residues of the NT, TM1 and the ECL1. They also localize in TM2 to a lesser extent (Fig. 3). In the case of Cx26, several mutations related to severe clinical phenotypes of KID are located at the transition zone between TM1 and the ECL1, a domain involved in both voltage gating and the control of HCs by extracellular Ca^2+^ [25]. Moreover, a cluster of syndromic mutations is found in the NT domain of the protein, which is involved in the fast gating of HCs [24, 130]. Nevertheless, a role of other regions on the regulation of HCs should be further considered. For example, the Cx32 mutation F235C, localized in the CT of the protein also induces HCs with gain of function [126].

The gain of HCs function has been also observed in Cx43 related connexinopathies, since mutations I31M (TM1), G138R (ICL) and G143S (ICL), all promotes gain of function (Table 3). As mentioned above, ICL is involved in regulation the fast V(j)-gating, which depend on the interaction with CT [28–32]. Moreover, Dobrowolski and co-workers (2008) [93] found an increased ATP-release in cultured cardiomyocytes from cardiac specific G138R-mutant mice. Interestingly, the authors proposed that HCs with gain of function in G138R-mutated cardiomyocytes might be one of the causes of arrhythmias.

As expected, some mutations induce loss of function HCs (Table 1 and Fig. 3). For example, mutations related to non-syndromic sensorineural hearing loss generate non-functional HCs [113]. Indeed, there are some syndromic mutations that exhibit non-functional HCs that only become gain of function when are co-expressed with their wild type partner or under aberrant interaction with Cx43 [13, 131].

Finally, It should be considering that in normal tissues cells could express several Cxs isoforms raising the possibility of interaction among Cxs isoforms. Recent results obtained in Dr. Martinez’ lab [13] and Dr. White’s group [131] suggest that the interaction between the mutated Cx and the co-expressed Cxs forming heterotypic/heteromeric channels needs to be taking into account to explain the clinical phenotypes of connexinopathies. Thus, interaction of mutants with wild type Cxs might ameliorate or worsen the clinical phenotypes. This possibility might augment when mutations affect critical segment involved in oligomerization compatibility, giving rise to aberrant heteromeric HCs, which makes pathological condition and effective treatment complex. In this scenario, further studies attempting to explore the pathological mechanism of connexinopathies should consider to study Cxs in heteromeric rather than homomeric states, which more closely resembles native cellular conditions.

## Conclusions

Most mutations causing connexinopathies generates total or partial loss of GJCs function. However, it is unclear if the severity of disease correlates with the level of GJCs loss of function. Mutations associated with loss of function GJCs are distributed along the entire protein sequence with no clear pattern of clustering at any segment, which suggest that GJC functionality is very sensitive to minor changes in Cxs protein, and that subtle changes in GJC functionality are sufficient to cause diseases. Less in known about the effect of mutations associated to connexinopathies on the functional state of HCs. The clearest correlation between gain of function HCs and disease has been found in most types of syndromic deafness associated to Cx26, in particular in KID syndrome. For others Cxs, few mutations are associated to gain of HCs function, however, we can not discard that this condition may be underestimated because most studies in the past have been more focused in GJCs than HCs. Therefore, it is yet difficult to make a general statement that represent all Cxs associated to connexinopathies. Nevertheless, it is clear that all mutations eliciting gain of HCs function are clustered in pore-associated domains like the NT and the TM1/ECL1, which are critical regions for gating and regulation.
